# Influence of Extra-Articular Augmentation on Clinical Outcomes and Survival in Patients Undergoing Anterior Cruciate Ligament Reconstruction: A Pseudorandomized Study

**DOI:** 10.3390/medicina61010116

**Published:** 2025-01-14

**Authors:** Antonio Maestro, Nicolás Rodríguez, Iván Pipa, Carmen Toyos, Lucía Lanuza, Filipe Machado, César Castaño, Santiago Maestro

**Affiliations:** 1 Hospital Begoña, 33204 Gijón, Spain; 2 Hospital Cruz Roja de Gijón, 33202 Gijón, Spain; rodriguezgcia@gmail.com; 3 Hospital Universitario de Cabueñes, 33394 Gijón, Spain; pipa85@msn.com (I.P.); lanuza.lucia@gmail.com (L.L.); 4 Hospital de Jarrio, 33795 Jarrio, Spain; carmentoyos@hotmail.com; 5 Centro Hospitalar de Setúbal, 2910-446 Setúbal, Portugal; efcmachado@gmail.com; 6 Clínica JLM, 33205 Gijón, Spain; cesarcastanofernandez@gmail.com; 7 Hospital Virgen de la Concha, 49022 Zamora, Spain; smaestro17@gmail.com

**Keywords:** ACL, anterior cruciate ligament, ALL, anterolateral ligament, knee, sports medicine, graft failure, pivot shift, return to sport

## Abstract

*Background and Objectives*: Up to 27% of anterior cruciate ligament (ACL) reconstruction cases result in a residual positive pivot shift sign, indicating anteroposterior and rotational instability. This instability can compromise returning to sports and increase the risk of further injuries. The biomechanical role of the anterolateral ligament (ALL) in controlling internal knee rotation is well known. However, there are no clinical trials comparing isolated ACL repairs to those combined with ALL reconstruction. Our objective is to compare the results of these techniques, with a primary focus on assessing knee stability and graft survival, to provide evidence for optimizing surgical approaches, particularly for athletes and physically active individuals. *Materials and Methods*: An observational study using paired score matching as a method of pseudo-randomization was conducted. Subjects were divided into an ACL group and an ACL+ALL group. Demographic and clinical variables were collected, as well as those related to complications and survival. *Results*: A total of 236 patients were included, which was reduced to 74 ACL and 37 ACL+ALL after pseudo-randomization, resulting in preoperatively comparable groups. During follow-up, differences in favor of ALL reinforcement were found on the pivot shift test (*p* = 0.007). No differences were found with regard to the Lachman test (*p* = 0.201), the International Knee Documentation Committee (IKDC) knee score (*p* = 0.169), the IKDC subjective score (*p* = 0.095), intensity of pain (*p* = 0.928), or complications (*p* = 0.529). Nor were differences found in the limb symmetry index; the single hop test (*p* = 0.710); the triple hop test (*p* = 0.653); the crossover hop test (*p* = 0.682); the 6 meter timed hop test (*p* = 0.360); the normalized Y-balance test (YBT) (*p* = 0.459 anterior; *p* = 0.898 posterolateral; and *p* = 0.211 posteromedial directions); or the limb symmetry index of the composite YBT (*p* = 0.488). There were no differences either with respect to return to sports practice (*p* = 0.723) or survival (*p* = 0.798). *Conclusions*: Patients treated by means of the ACL+ALL technique obtained higher rotational stability, as measured by the pivot shift test, than those subjected to an isolated ACL repair. No differences were found with respect to Lachman test, complications, IKDC, pain, or survival.

## 1. Introduction

The overarching aim of anterior cruciate ligament (ACL) reconstruction surgery is to restore knee stability and allow patients to resume their routine activities, reducing the risk of cartilage degeneration [1]. ACL reconstruction surgery protects the medial meniscus [2,3,4], minimizing the risk of arthritic knee degeneration [5,6,7,8,9,10,11,12]. However, for surgical ACL reconstruction to be successful, the surgeon must carefully manage the knee’s anteroposterior and, particularly, rotational laxity [13], as up to 27% of intra-articular ACL reconstruction cases were reported to result in a residual positive pivot shift sign [13,14,15,16,17,18].

The pivot shift phenomenon is typically indicative of a combination of anteroposterior and rotational instability [13], which could prevent participation in pivoting sports and result in failure of the reconstruction or in injuries to cartilage or the menisci [14]. Although a positive pivot shift sign was traditionally considered to be tantamount to an ACL injury, the truth is that this sign is typically observed when both the ACL and the knee’s peripheral structures are damaged [13,16,19,20,21]. In fact, some isolated injuries to these peripheral structures often present with a pivot shift sign in the absence of an ACL injury, and not all ACL lesions result in a pivot shift sign [13,20,21].

**Figure 1:**
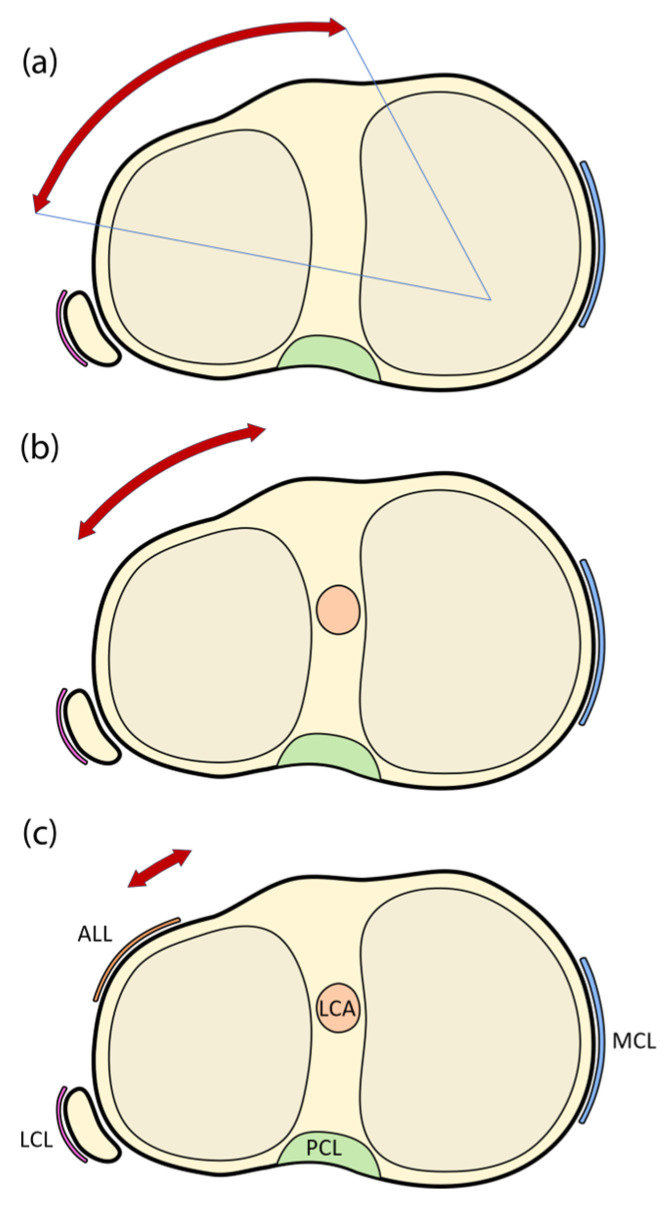
Schematic representation showing instances of knee joint instability, with the red arrows indicating the residual rotational instability. (**a**) without anterior cruciate or anterolateral ligament; (**b**) with anterior cruciate ligament but without anterolateral ligament; and (**c**) with all articular structures present. ALL: anterolateral ligament; LCA: anterior cruciate ligament; PCL: posterior cruciate ligament; MCL: medial collateral ligament; LCL: lateral collateral ligament.

The torsional laxity that results from some intra-articular reconstructions together with the well-known biomechanical role played by the anterolateral ligament (ALL) in controlling internal knee rotation [22,23,24,25,26,27,28,29,30,31,32,33,34,35] have understandably led to a renewed interest in the use of lateral extra-articular tenodeses to improve the results of ACL repair procedures [36,37,38]. As they are located in the periphery of the knee, ALL-augmented reconstructions provide a more efficient lever arm for controlling internal tibial rotation compared to isolated reconstructions (Figure 1) [13]. Indeed, the Anterolateral Ligament Expert Group determined that the main function of the ALL is to restrict internal tibial rotation, which may affect pivot shift in cases of ACL insufficiency [35].

Although intra- and extra-articular reconstruction techniques were, in the past, used in isolation, combined reconstructions of the ACL and the ALL act synergistically, making it possible to control anteroposterior and rotational laxity [14,39,40,41,42,43,44,45,46,47], protecting the graft during the biological healing phase, and offering an additional restraint against pivot shift instability. Indeed, an in vitro study showed that extra-articular reconstructions are able to reduce the forces borne from an intra-articular graft by 43% [13].

However, despite the biomechanical advantages of ALL reinforcement, its clinical implications remain controversial. Some studies suggest that ALL reinforcement may accelerate degeneration of the lateral compartment [48,49], while others attribute such outcomes to outdated surgical techniques, excessive tensioning, or inadequate postoperative care. Furthermore, many studies lack long-term follow-ups or present conflicting results regarding stability and survival rates [50,51]. These gaps in the literature limit the ability to draw definitive conclusions about the clinical benefits and risks of ALL reinforcement.

Given the novelty of this technique, there are no clinical trials comparing the outcomes of isolated ACL repairs with those of an ACL repair combined with an extra-articular ALL reconstruction. The purpose of the present study is to compare both techniques, as well as to analyze the survival and complications rates associated with each of them.

## 2. Materials and Methods

This study was approved by the Ethics Committee for Drug Research of the Principality of Asturias on 16 March 2020 (code: 2020.111). All subjects gave their informed consent prior to inclusion.

The study analyzed all patients subjected to an ACL repair (isolated or combined with an ALL reconstruction) from 1 January 2000 to 31 December 2021, who agreed to participate in the study. Since the objective of our analysis was to assess a new treatment strategy, a single-arm before-and-after study design was chosen [52,53]. Prior to 2018, all cases were treated with isolated ACL repair, while ALL augmentation was introduced from that year forward. Subjects were divided into one of the following groups:

− ACL group: Isolated intra-articular arthroscopic reconstruction of the ACL.
− ACL+ALL group: The intra-articular reconstruction was augmented by an extra-articular reinforcement that stemmed from the lateral aspect of the femur, slightly proximal and posterior to the epicondyle, and attached distally to the tibia, 15 mm below the joint line and half way between the fibular head and Gerdy’s tubercle [14,54,55,56,57].

The variables analyzed included the demographic data (age, sex, height, weight, etc.); the mechanism of injury; comorbidities and previous surgeries; Lachman test [58]; the pivot shift test [59]; Tegner score [60]; range of motion; the type of surgery (isolated ACL repair or ACL repair reinforced with a repair of the ALL); the type of repair; tunnel and graft thickness; associated maneuvers; complications; pain, as measured by the Visual analog scale (VAS) [61]; failed repairs; hop test (simple, triple, crossover and 6 meter times tests) [62]; the Y-balance test (YBT) [63,64,65,66]; the International Knee Documentation Committee (IKDC) subjective score [67]; and the IKDC knee score. All the data, except for functional scores, was collected both preoperatively and in the course of follow-up.

### 2.1. Anatomy and Biomechanics of ALL

Although not the primary focus of this study, a brief overview of the anatomy and biomechanics of the anterolateral ligament (ALL) is provided. Recent research suggests the ALL is a distinct ligament in the anterolateral portion of the knee, attaching near the lateral epicondyle of the femur. Its distal insertion point is located between the fibular head and Gerdy’s tubercle [24,25]. Biomechanical studies show that the length of the ALL increases with knee flexion and tibial internal rotation, suggesting it plays a key role in resisting internal rotation. The ALL acts as a secondary stabilizer, particularly in ACL-deficient knees, by restricting tibial internal rotation and influencing the pivot shift test [24,35].

### 2.2. Statistical Analysis

Given that the goal of our study is to evaluate a new treatment strategy and that all patients were initially treated with isolated ACL reconstruction, with the addition of ALL reinforcement introduced later, randomization was not feasible [52]. In this context, propensity score matching (PSM) using nearest neighbor matching with a 2:1 ratio was conducted. This approach consolidates multiple covariates into a single scalar function, enabling the comparison of subjects with similar characteristics and improving the validity of treatment effect estimates [68,69]. Once paired, a descriptive analysis of the data was performed using measures of central tendency and dispersion. Either parametric or non-parametric tests of mean differences were used to make comparisons between the groups, depending on the normality of the samples. Qualitative variables were analyzed using Pearson’s chi-squared test or the Fisher Exact Test, depending on the magnitude of the expected values. Survival was analyzed by means of the Kaplan–Meier method, using failure for any cause as an endpoint. All cases, and not only the paired ones, were included in the survival analysis to avoid bias. Differences in survival were evaluated using a log rank test. Statistical significance was set, in all cases, at a *p*-value of 0.05. The data were analyzed using R software (R Development Core Team), version 4.1.3 [70]. Propensity score matching was applied using the MatchIt package [71].

## 3. Results

**Figure 2:**
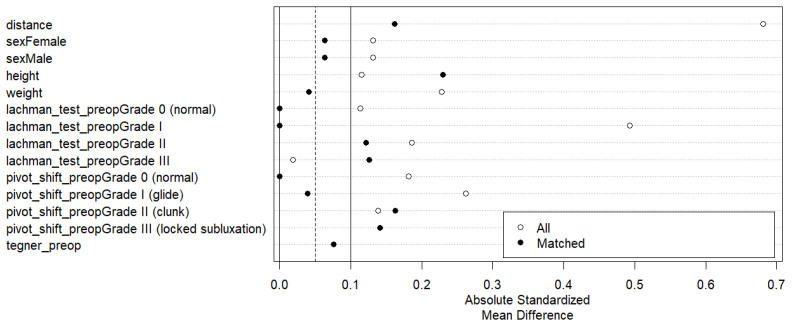
Love plot showing reduction in absolute standardized mean difference among groups after application of paired score matching method.

The study included 236 patients subjected to ACL reconstruction surgery, of whom 199 (84.3%) were included in the isolated ACL repair group and 37 (15.7%) in the ALL augmentation group (ACL+ALL). After PSM, 37 patients were retained in the ACL+ALL group (33.3%) and 74 (66.6%) in the ACL group. Figure 2 shows a significant reduction in the absolute standardized mean difference, increasing comparability between the groups.

### 3.1. Anthropometry and Diagnosis

The anthropometric and diagnostic data obtained (Table 1) showed that both groups were similar in terms of sex distribution (78% of males in the ACL group vs. 76% in the ACL+ALL group, *p* = 0.748); laterality (45% right legs in the ACL group vs. 51% in the ACL+ALL group, *p* = 0.542); age (30.9 years in the ACL group vs. 30.2 in the ACL+ALL group, *p* = 0.781); height (171.2 cm in the ACL group vs. 173.4 in the ACL+ALL group, *p* = 0.927); and weight (73.2 kg in the ACL group vs. 72.3 in the ACL+ALL group, *p* = 0.714). Mean length of follow-up was, however, considerably longer in patients in the isolated reconstruction group (90.1 months vs. 29.9 months in the ACL+ALL group, *p* < 0.001).

### 3.2. Preoperative Evaluation

**Figure 3:**
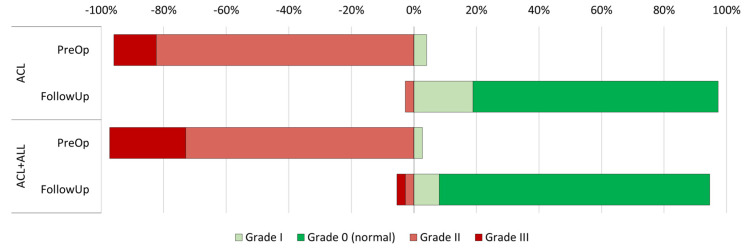
Representation of progression of Lachman test results in both groups between preoperative and postoperative period.

**Figure 4:**
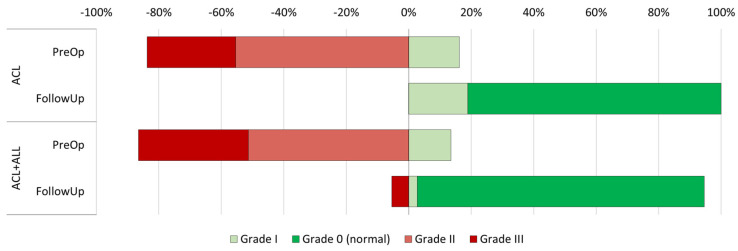
Representation of progression of pivot shift test results in both groups between preoperative and postoperative period.

The preoperative clinical evaluation (Table 2) did not reveal statistically significant differences between the groups in terms of flexion (108.6 degrees in the ACL group vs. 113.5 in the ACL+ALL group, *p* = 0.169) or extension (−3.6 degrees in the ACL group vs. −3.8 in the ACL+ALL group, *p* = 0.747) of the injured limb. In addition, anteroposterior knee stability, as measured by the Lachman test, was also similar in both groups (*p* = 0.350) (Figure 3). Nor was statistical significance achieved (*p* = 0.756) with respect to rotational stability, as measured by the pivot shift test (Figure 4).

### 3.3. Surgical Information

All the information about the surgical procedure is contained in Table 3. All repairs in this study were performed using the semitendinosus and the gracilis. No statistically significant differences were found between the groups with respect to the percentage of meniscus repairs (61% in the ACL group vs. 65% in the ACL+ALL group, *p* = 0.678) or chondral repairs (4% in the ACL group vs. 11% in the ACL+ALL group, *p* = 0.219) performed. Similarly, although the percentage of intraoperative complications was higher in the group without ALL reinforcement, the difference did not reach statistical significance (4% in the ACL group vs. 0% in the ACL+ALL group, *p* = 0.299).

### 3.4. Evaluation During Follow-Up Period

Table 4 and Table 5 describe the clinical assessment carried out during the follow-up period. No statistically significant or clinically relevant differences were found between the groups in terms of knee extension (*p* = 0.191). However, a difference was observed in flexion (133.2° in ACL vs. 129.6° in ACL+ALL; *p* = 0.037), although this difference is small and lacks clinical relevance, as indicated by the effect size (Cohen’s d = 0.425).

The Lachman test did not find differences during the follow-up period (Figure 2) (*p* = 0.201). Contrarily, the rotational stability achieved by patients with an anterolateral reinforcement, as measured by the pivot shift test, was superior to that achieved by the group where the standard technique was employed (92% grade in the ACL+ALL group vs. 81% in the ACL group, *p* = 0.007) (Figure 3).

Results on the IKDC knee score were similar among groups (*p* = 0.169) (Figure 4). Also, the differences observed in the IKDC subjective score were not statistically significant (82.4% in the ACL group vs. 83.0% in the ACL+ALL group; *p* = 0.095). No differences were observed with respect to pain, as measured by VAS (0.70 in the ACL group vs. 0.68 in the ACL+ALL group, *p* = 0.928), or the percentage of patients experiencing complications in the course of the follow-up (9% in the ACL group vs. 14% in the ACL+ALL group, *p* = 0.529).

No statistically significant differences were observed in any of the hop tests evaluated using the limb symmetry index (LSI). Indeed, the difference between the groups was 95.3% for the ACL group vs. 94.2% for the ACL+ALL group (*p* = 0.710) in the single hop test; 96.4% for the ACL group vs. 98.0% for the ACL+ALL group (*p* = 0.653) in the triple hop test; 94.6% for the ACL group vs. 95.8% for the ACL+ALL group (*p* = 0.682) in the crossover hop test; and 102.7% for the ACL group vs. 105.5% for the ACL+ALL group (*p* = 0.360) in the 6 meter timed hop test.

No differences were found between the groups with respect to the various YBT measures, as normalized to the subjects’ leg length, when comparing the differences between the healthy and the injured leg in the three directions of space (*p* = 0.459 for the anterior direction; *p* = 0.898 for the posterolateral direction; and *p* = 0.211 for the posteromedial direction), or to the LSI of the composite YBT (*p* = 0.488).

### 3.5. Return to Sports Practice

The proportion of patients who were able to return to the same or a higher level of sports performance was similar in both groups (83.8% in the ACL group vs. 81.1% in the ACL+ALL group, *p* = 0.723) (Table 6).

No differences were found when comparing the subgroups traditionally associated with a lower rate of return to sports practice such as women (78.3% for women vs. 84.1% for men, *p* = 0.539) or subjects with a meniscus lesion (81.0% for subjects without a meniscus injury vs. 84.1% for subjects with a meniscus injury, *p* = 0.674) (Table 7).

### 3.6. Survival

**Figure 5:**
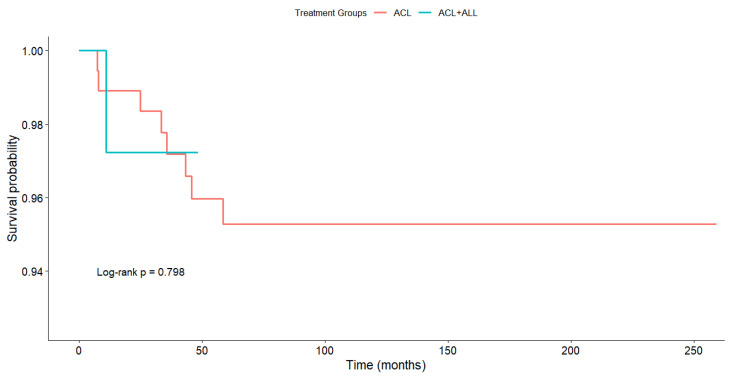
Estimated survival according to Kaplan–Meier method.

Survival, as estimated by the Kaplan–Meier method, was 95.3% in the ACL group and 97.2% in the ACL+ALL group, with no statistically significant differences being found between the groups on the log rank test (*p* = 0.798) (Figure 5).

## 4. Discussion

The most significant finding of the present study is that ACL repairs that are augmented by restoration of the ALL result in greater knee stability, as measured by the pivot shift test. In the following section, we shall provide a detailed analysis of our results.

### 4.1. Pivot Shift Test and Lachman Test

The pivot shift test [59] is recognized as an important tool to measure clinical outcomes following an ACL repair [10,72,73]. Several studies have confirmed that the presence of a positive pivot shift sign is indicative of instability and poorer objective and subjective results, as well as of an impossibility to return to preinjury levels of sports performance [74,75]. Under this perspective, given that the extra-articular augmentation of ACL repairs prevents tibial plateau subluxations and protects the intra-articular graft [76,77,78] and that various studies have demonstrated that intra- and extra-articular repairs act synergistically in controlling the pivot shift phenomenon [14,39,40,41,42,43,44], it is only logical to expect the ACL+ALL group to be more capable of overcoming this kind of instability. It must be said though that some authors have argued that ALL augmentations can only limit pivot shift instability at knee positions around 90 degrees of flexion [79].

The present series provides confirmation of the hypothesis above, as we found a higher percentage of patients without pivot shift instability (grade 0) in the ACL+ALL group (92% in the ACL group vs. 81% in the ACL+ALL group, *p* = 0.007). Our findings are in line with those of Song et al. [47], who found a mean positive pivot shift prevalence of 27.2% in their ACL group (26% in our series) and 13.3% in their ACL+ALL group (8% in our series), indicating a clear superiority of ALL reinforcement. The results reported in the literature are somewhat inconsistent. The study with the longest follow-up to date [80] was also unable to find statistically significant differences between the groups with respect to the presence of a residual pivot shift sign (*p* = 0.230), although its authors—like us—consider that patients in the ALL reinforcement group are able to control their residual pivot shift instability more effectively. Similarly, Hewison et al. [81] performed a systematic review where they analyzed eight articles of which three did not find any pivot shift differences between the groups analyzed, four found differences in favor of ALL reinforcement, and one found differences in favor of the isolated ACL reconstruction. An analysis of 70 professional athletes [82] obtained better results in terms of pivot shift reduction (5.7% positive cases), although the reported reoperations and failure rates were considerably higher (15.7% and 5.7%, respectively).

As far as the Lachman test is concerned, we did not find differences among the groups (*p* = 0.201). This is in line with the fact that ALL reinforcement was not found to exert a very marked effect on anteroposterior knee stability.

### 4.2. Return to Sports Practice

The return-to-sports rates reported in the literature on ACL repairs are highly heterogeneous. A systematic review of 7556 cases found that only 65% of patients were able to go back to their preinjury activity levels [83], whereas another one, which only included patients operated using extra-articular augmentation techniques combined with an intra-articular repair [84], reported rates between 64% and 100% but failed to provide a mean value. Other authors found considerably lower return-to-sports rates [85] (44.2% for patients with an isolated ACL repair and 52% for those with combined ACL+ALL repairs). Overall, a majority of authors appear to be more favorable to ALL reinforcement, even if practically none of the studies achieved statistical significance [86,87]. This evidence is consistent with our data.

Some of the factors influencing the likelihood for a patient to go back to preinjury activity levels are sex, the presence or absence of meniscal damage, and the type of graft used [10,36]. Our study found no differences in the rate of return to preinjury levels of activity between sexes (*p* = 0.539), or in the presence or absence of concomitant meniscal damage (*p* = 0.674). The role of the type of graft used could not be evaluated because the same type of repair was performed on all the patients in this study.

### 4.3. IKDC and Pain

Most studies in the literature have found no association between patient-reported outcomes and the use, or otherwise, of ALL reinforcement [36,47,80,85,86,88]. Our study was no different in that regard as neither the IKDC subjective score nor VAS, used to measure the intensity of pain, found any statistically significant or clinically relevant differences between the groups (*p* = 0.095 and *p* = 0.928, respectively). Helito et al. [89] did find statistically significantly different clinical results, as measured by the IKDC subjective score, when applying the ACL+ALL technique to patients where over 12 months had elapsed between the injury and the repair (87.1 for the ACL group vs. 92.7 for the ACL+ALL group, *p* = 0.001). This may be due to the marked anterolateral knee laxity observed in these patients with chronic ACL insufficiency. In this regard, our proportion of chronic patients was insufficient to verify this observation. Sonnery-Cottet et al. [86] found statistically significant differences in favor of an isolated ACL repair on the KOOS pain subscale. Nonetheless, these authors do not consider the difference to be clinically relevant.

### 4.4. YBT and Hop Tests

Interlimb asymmetries in YBT were used to estimate the risk of sustaining an injury in the lower limbs [64,90,91] and to evaluate the outcome of surgical procedures in the lower extremities, including ACL repair [92,93,94]. When administering YBT, we used reach distances normalized to limb length, which allowed us to combine males and females in the same analysis, as it was shown that differences between the sexes disappear when results are normalized to limb length [95].

Our analysis did not find statistically significant asymmetries between the groups in any of the three dimensions of space analyzed or in the composite scores. Moreover, we did not find any of the YBT parameters evaluated in our study to fall outside the mean lateral asymmetry value for the general population, which was reported to range between 3% and 8% [64]. Similarly, we found no differences in the limb symmetry index between any of the hop tests analyzed, which is in line with previous findings [88].

### 4.5. Survival

Analyzing survival is no easy task given the plethora of factors affecting the survival of ACL reconstruction [10,96,97] and the high variability of the reported failure rates, which range from 1.4% to 28% in high-risk patients [36,98,99]. In addition, few published articles look into the influence of ALL reinforcement on the rupture of ACL repairs [36]. In our case, survival, as estimated by the Kaplan–Meier method, did not exhibit any statistically significant differences between the two reconstruction techniques (*p* = 0.798). In a study of 502 patients where they compared three techniques including ALL reinforcement, Sonnery-Cottet et al. [36] did find a correlation between the rupture rate and the technique used, with a rupture rate of 4.13% in the hamstring tendon (HT)+ALL group, 10.77% in the quadrupled hamstring tendon graft (4HT) group, and 16.77% in the bone–patellar tendon–bone graft (B-PT-B) group.

### 4.6. The Strengths and Limitations of the Study

The present study is not exempt from limitations. The most obvious one has to do with its retrospective and non-randomized nature. However, before-and-after designs, which are usually interventional rather than observational, are justified in situations where it would be deemed unethical to withhold essential treatment from patients in the control group, as is the case here [52,53]. Additionally, these designs may offer a high level of generalizability [100]. Also, PSM is a valuable way to control for bias and achieve pseudo-randomization in retrospective observation studies [68,69]. Other limitations include a failure to analyze the progression of osteoarthritis, which would have been desirable as techniques such as ALL+ACL reconstruction were blamed for increasing the risk of developing the disease. Nevertheless, in these circumstances, where there are no randomized clinical trials comparing these two treatments–and, given the ethical limitations, we believe there will not be any—this comparison using PSM provides a valuable opportunity to understand the effects of anterolateral reinforcement in ACL repair.

## 5. Conclusions

Patients treated by means of the ACL+ALL technique obtained higher rotational stability, as measured by the pivot shift test, than those subjected to an isolated ACL repair. No differences were found with respect to Lachman test, complications, IKDC, pain, or survival. Therefore, and given that the anterolateral reinforcement is a surgical gesture with low morbidity, it would be advisable to perform it as a complement in all surgeries where residual instability is suspected.

## Figures and Tables

**Table 1:** Anthropometric and diagnostic data.

Variable	N (%)	Mean	SD	*p*-Value
Sex (M/F)				
ACL	58 (78%)/16 (22%)	-	-	0.748
ACL+ALL	28 (76%)/9 (24%)	-	-	
Laterality (R/L)				
ACL	33 (45%)/40 (55%)	-	-	0.542
ACL+ALL	19 (51%)/18 (49%)	-	-	
Age				
ACL	-	30.9	11.9	0.781
ACL+ALL	-	30.2	15.1	
Height				
ACL	-	171.2	8.4	0.927
ACL+ALL	-	173.4	9.6	
Weight				
ACL	-	73.2	10.8	0.714
ACL+ALL	-	72.3	12.1	

**Table 2:** Preoperative clinical evaluation.

Variable	N (%)	Mean	SD	*p*-Value
Injured limb—Flexion (°)				
ACL	-	108.6	14.1	0.169
ACL+ALL	-	113.5	19.1	
Injured limb—Extension (°)				
ACL	-	−3.6	3.1	0.747
ACL+ALL	-	−3.8	4.3	
Lachman test—PreOp				
ACL				0.350
Normal	0 (0%)	-	-	
Grade I	3 (4%)	-	-	
Grade II	61 (82%)	-	-	
Grade III	10 (14%)	-	-	
ACL+ALL				
Normal	0 (0%)	-	-	
Grade I	1 (3%)	-	-	
Grade II	27 (73%)	-	-	
Grade III	9 (24%)	-	-	
Pivot shift—PreOp				
ACL				0.756
Grade 0 (normal)	0 (0%)	-	-	
Grade I (glide)	12 (16%)	-	-	
Grade II (clunk)	41 (55%)	-	-	
Grade III (locked subluxation)	21 (28%)	-	-	
ACL+ALL				
Grade 0 (normal)	0 (0%)	-	-	
Grade I (glide)	5 (14%)	-	-	
Grade II (clunk)	19 (51%)	-	-	
Grade III (locked subluxation)	13 (35%)	-	-	

**Table 3:** Surgical information.

Variable	N (%)	Mean	SD	*p*-Value
Meniscal repair				
ACL	45 (61%)	-	-	0.678
ACL+ALL	24 (65%)	-	-	
Chondral repair				
ACL	3 (4%)	-	-	0.219
ACL+ALL	4 (11%)	-	-	
IntraOp complications				
ACL	4 (5%)	-	-	0.299
ACL+ALL	0 (0%)	-	-	

**Table 4:** Clinical evaluation at follow-up (I).

Variable	N (%)	Mean	SD	*p*-Value
Injured limb—Flexion (°)				
ACL	-	133.2	7.7	0.037 *
ACL+ALL	-	129.6	9.8	
Injured limb—Extension (°)				
ACL	-	−0.1	0.8	0.191
ACL+ALL	-	−0.7	2.4	
Lachman test—Follow-Up				
ACL				0.201
Normal	58 (78%)	-	-	
Grade I	14 (19%)	-	-	
Grade II	2 (2%)	-	-	
Grade III	0 (0%)	-	-	
ACL+ALL				
Normal	32 (86%)	-	-	
Grade I	3 (8%)	-	-	
Grade II	1 (3%)	-	-	
Grade III	1 (3%)	-	-	
Pivot shift—Follow-Up				
ACL				0.007 *
Grade 0 (normal)	60 (81%)	-	-	
Grade I	14 (19%)	-	-	
Grade II	0 (0%)	-	-	
Grade III	0 (0%)	-	-	
ACL+ALL				
Grade 0 (normal)	34 (92%)	-	-	
Grade I	1 (3%)	-	-	
Grade II	0 (0%)	-	-	
Grade III	2 (5%)	-	-	
IKDC Knee score—Follow-Up				
ACL				0.169
A—Normal	70 (95%)	-	-	
B—Nearly normal	4 (5%)	-	-	
C—Abnormal	0 (0%)	-	-	
D—Severely abnormal	0 (0%)	-	-	
ACL+ALL				
A—Normal	35 (97%)	-	-	
B—Nearly normal	0 (0%)	-	-	
C—Abnormal	1 (3%)	-	-	
D—Severely abnormal	0 (0%)	-	-	
IKDC Subjective Score—Follow-Up				
ACL	-	82.4	5.8	0.095
ACL+ALL	-	83.0	9.1	
Pain—VAS				
ACL	-	0.70	1.3	0.928
ACL+ALL	-	0.68	1.2	
Complications During Treatment				
ACL	7 (9%)	-	-	0.529
ACL+ALL	5 (14%)	-	-	

* Statistically significant differences have been detected between the compared groups.

**Table 5:** Clinical evaluation at follow-up (II).

Variable	Mean	SD	*p*-Value
LSI—Single hop test			
ACL	95.3	15.4	0.710
ACL+ALL	94.2	13.5	
LSI—Triple hop test			
ACL	96.4	12.9	0.653
ACL+ALL	98.0	20.2	
LSI—Crossover hop test			
ACL	94.6	15.0	0.682
ACL+ALL	95.8	13.9	
LSI—6 meter timed hop test			
ACL	102.7	12.2	0.360
ACL+ALL	105.5	15.3	
LSI—YBT composite score (normalized)			
ACL	100.0	12.6	0.488
ACL+ALL	98.4	10.2	

**Table 6:** Evolution of Tegner score (return to physical activity) by groups.

Return to Sports	ACL	ACL+ALL	*p*-Value
Same or better level	83.8%	81.1%	0.723
Worse level	16.2%	18.9%	

**Table 7:** Evolution of Tegner score (Return to physical activity). By other factors.

Group	Same or Better Level	Worse Level	*P*-Value
Sex			
Males	84.1%	15.9%	0.539
Females	78.3%	21.7%	
Meniscal damage			
No	84.1%	15.9%	0.674
Yes	81.0%	19.0%	

## Data Availability

Datasets are available on request from the authors.

## Abbreviations

The following abbreviations are used in this manuscript:

ACL	Anterior cruciate ligament
ALL	Anterolateral ligament
ACL+ALL	Anterior cruciate ligament repair combined with anterolateral ligament reconstruction
PCL	Posterior cruciate ligament
MCL	Medial collateral ligament
LCL	Lateral collateral ligament
YBT	Y-balance test
IKDC	International Knee Documentation Committee
PSM	Propensity score matching
VAS	Visual analog scale for pain
